# The Neglected Factor in the Relationship between Executive Functioning and Obesity: The Role of Motor Control

**DOI:** 10.3390/healthcare10091775

**Published:** 2022-09-15

**Authors:** Marco La Marra, Antonietta Messina, Ciro Rosario Ilardi, Giuseppe Verde, Raffaella Amato, Nadia Esposito, Simona Troise, Antonella Orlando, Giovanni Messina, Vincenzo Monda, Girolamo Di Maio, Ines Villano

**Affiliations:** 1 Department of Experimental Medicine, University of Campania “Luigi Vanvitelli”, 80138 Naples, Italy; 2 Department of Psychology, University of Campania “Luigi Vanvitelli”, 81100 Caserta, Italy; 3 Neurological Unit, CTO Hospital, AORN “Ospedali dei Colli”, 80131 Naples, Italy; 4 Department of Clinical and Experimental Medicine, University of Foggia, 71122 Foggia, Italy; 5 Department of Movement Sciences and Wellbeing, University of Naples “Parthenope”, 80133 Naples, Italy

**Keywords:** obesity, executive functions, BMI, waist circumference, motor performance

## Abstract

Background: The association between obesity and executive functions (EFs) is highly controversial. It has been suggested that waist circumference (WC), compared to body mass index (BMI), is a better indicator of fat mass and EFs in obesity. Moreover, according to the viewpoint that the brain’s functional architecture meets the need for interactive behavior, we hypothesize that the relationship between EFs and body weight might be mediated by the motor performance. Methods: General executive functioning (frontal assessment battery-15), additional cognitive subdomains (trail making test and digit span backward), and motor performance (finger tapping task) were assessed in a sample that included 330 volunteers (192 females, M age = 45.98 years, SD = 17.70, range = 18–86 years). Results: Hierarchical multiple regression analysis indicated that the FAB15 score and FTT negatively predicted WC but not BMI. A subsequent mediation analysis highlighted that the indirect effect of FAB15 on WC through finger tapping was statistically significant. Conclusions: Our results suggest that WC, as compared to BMI, is a more effective measure for studying the association between EFs and body weight. Still, we found that the motor domain partially mediates the dynamics of such a relationship.

## 1. Introduction

In recent decades, a growing number of studies have investigated the relationship between obesity and cognition [1,2,3,4]. In particular, special emphasis was given to executive functions (EFs) [5,6]. Traditionally, they are considered a family of top–down cognitive processes (e.g., attention, processing speed, set-shifting, inhibitory control, working memory, concept formation, planning) supporting forethought and goal-directed actions [7,8,9]. In particular, EFs allow individuals to cope adaptively with environmental demands, especially in unfamiliar and conflictual contexts [7,9] through inhibition of dominant responses, blunting interfering stimuli, or resisting temptations [10].

Interestingly, it has been suggested that differences in EFs may predict body weight variability [11]. For instance, decreased EFs have been associated with reduced food intake inhibition [12,13,14], high-fat food ingestion [15], lower appetite regulation [16], decreased energy expenditure [17], increased emotional eating [18], inability to learn from past experiences [19], delayed weight loss [12], poor adherence to healthy nutrition [20,21], and poor outcomes to weight loss interventions [22]. In line with these findings, some studies have found poor executive performance in patients with obesity [11,23,24,25]; furthermore, neuroimaging evidence exists on the relationship between decreased neural activity within the frontal cortex—the main cortical substrate of EFs—and obesity [26,27,28]. This evidence might be supported by studies finding a significant relationship between adipose tissue and systemic inflammation interacting with the physiology of the blood–brain barrier, likely affecting cognitive performance [29,30,31,32,33].

Nevertheless, it is important to stress that conflicting results are also available. Indeed, some studies have reported that obese subjects show equal or better executive performance than normal-weight subjects [34,35,36,37,38,39,40,41]. These latter observations, while counterintuitive, do concur with the phenomenon known as the “obesity paradox” [42,43,44,45], which refers to the possible positive association between obesity and health outcomes, especially in the elderly population.

Reiterating what has been stated in previous research [11,41,46], we argue that the above-mentioned inconsistencies could be attributed to several factors, including the assessment techniques employed to measure obesity, the presence of confounding covariates contaminating the relationship between cognitive performance and obesity, and the inhomogeneous methods used to assess EFs. Furthermore, few studies exploring the relationship between obesity and EFs are longitudinal; therefore, many questions on the direction of such a relationship, i.e., on the causality between obesity and EFs, remain unanswered [47].

Although body mass index (BMI) is currently the most widely used obesity index, it has received several criticisms [48,49]. Among them, BMI does not discriminate between muscle and adipose tissues and cannot directly assess regional adiposity [50]. Moreover, the use of BMI as a measure of obesity results in estimation biases involving obesity-related effects [49]. Alternatively, it has been suggested that waist circumference (WC) should be preferred to BMI as it proved to be a measure more sensitive to visceral obesity. Moreover, WC is a reliable predictor of several health risk factors, such as cardiovascular disease, diabetes, and increased mortality [51,52,53]. Further, it has been suggested that WC is a better indicator than BMI for studying the relationship between obesity and cognitive functions [46].

In addition, many studies did not disclose the role exerted by relevant covariates characterizing the study sample. For instance, obesity is often accompanied by a number of comorbidities, such as high blood pressure, diabetes, and metabolic syndrome, which are clinical conditions that may independently affect cognition [54,55,56]. Still, sociodemographic variables (sex, age, education) are strictly related to cognitive performance and their role is often overlooked [57]. This lack of transparency and methodological rigor makes it unclear whether obesity predisposes to cognitive impairment, or vice versa, because of the possible confounding effects of covariates.

Finally, the relationship between obesity and executive functioning could be mediated by the motor performance, a variable poorly addressed in the neuropsychological literature. Indeed, although some cognitive tasks investigating EFs are based on a verbal response set (e.g., verbal fluency, Stroop test), most executive tests require hand motor responses to be properly performed. Furthermore, it is well-known that motor and executive domains share common neural mechanisms [58,59].

Theoretically speaking, the sensorimotor interaction model by Koziol and Lutz [60,61] may further explain the link between EFs and the motor domain. Although EFs are traditionally considered top–down mental processes, the functional architecture of the brain evolved from childhood to adulthood in order to meet the needs of interactive behavior. This would generate both procedural and declarative knowledge supporting executive functioning [61]. For instance, the fronto-parieto-cerebellar network is involved in the detection of discrepancies between expected and actual sensorimotor feedback. This mechanism has considerable evolutionary relevance, as it may be used to correct future predictions and allows more effective interaction with the environment [62]. Moreover, the above network interacts with basal ganglia to plan and control motor sequences, as well as to predict their resulting outcomes [61,62].

There is some evidence highlighting an inverse relationship between body weight and speed or dexterity during movement execution [63,64,65,66]. Adiposity has been related to the muscle quality ratio that is associated, in turn, to hand strength [67] and motor conduction speed during gross repetitive hand movements [68]. Obese children and adolescents would show reduced motor skills, e.g., fine and gross motor control, and delayed motor development [69,70,71,72,73,74,75,76,77,78,79,80]. In middle-aged and older adults, higher adiposity levels seem to be correlated with lower motor speed and manual dexterity [81]. More generally, in controlling movements, obese individuals would invest greater attentional resources to compensate for their motor deficits [82,83]. In line with these behavioral observations, individuals with obesity appear to show reduced motor cortex plasticity as compared to normal weight controls [84]. Structural brain mapping studies identified three neural networks strongly related to body weight, i.e., the default mode network, the fronto-executive network, and the fronto-parietal network. Interestingly, recent evidence has suggested that the default mode network’s functional connectivity significantly mediated the relationship between body weight and executive functioning during the execution of the finger tapping, a simple motor task requiring subjects to perform as many finger taps as possible within a given time unit [85].

In line with the above, the current cross-sectional study sought to re-examine the relationship between obesity and EFs. Based on previous research [46], we hypothesize that WC is a more sensitive measure than BMI in capturing executive blunting. Moreover, we hypothesize that the relationship between EFs and body weight could be mediated by gross motor function evaluated by the finger tapping task. In this regard, obesity seems to undermine both motor and frontal/executive abilities [25,26,27,28,46,63,64,65,66]. In addition, in line with the sensorimotor interaction model [60,61], the integrity of the executive processes might depend on bottom-up interactive processes supported by motor behavior. Importantly, the potential confounding effects of sociodemographic covariates were taken into account.

## 2. Materials and Methods

### 2.1. Participants

Participants were 365 (212 females) Italian volunteers recruited through a convenience sampling method across different districts of the Campania, Calabria, and Puglia regions (Southern Italy). Eligible participants met the following inclusion criteria: age ≥ 18 years, formal education ≥ 5 years (i.e., primary school), and adjusted score greater than or equal to 23.8 on the mini-mental state examination (MMSE) [86]. According to normative datasets, deficient scores on the administered cognitive tests (see materials and procedure) were considered additional exclusion criteria. Exclusion criteria were: previous or current history of intellectual and/or linguistic deficits, neurocognitive, psychiatric, or psychopathological disorders, and presence of serious health conditions, including cancer and severe obesity. No participant had a history of alcohol or substance abuse/addiction. Participants were not treated with drugs interfering with cognitive processes. Individuals with pharmacologically-compensated chronic medical illnesses, such as hypertension, type II diabetes, or cardiovascular diseases were not excluded to prevent the construction of a hyper-normal sample. According to the exclusion criteria, data from 35 individuals were excluded from the dataset (final *N* = 330).

### 2.2. Materials and Procedure

Testing was performed individually in a soundproof room. After sociodemographic data acquisition (e.g., sex, age, and years of formal education), anthropometric measurements, i.e., weight, height, and WC, were measured. BMI values were calculated according to Quetelet’s formula (kg/m^2^). WC was measured by placing a measuring tape in a horizontal plane around the abdomen, 2 cm above the belly button. Following the administration of MMSE, participants were administered a brief neuropsychological assessment protocol including the frontal assessment battery-15 [87], the trail making test [88], the digit span backward [89], and the finger tapping task [90].

*Frontal assessment battery-15 (FAB15)*. The FAB15 is a screening battery devised to assess general executive/frontal functions. It consists of five subtests exploring abstraction abilities, generativity, planning, sensitivity to interference, and inhibitory control. Three out of the five subtests are based on a motor response set while the remaining ones require a verbal response [87,91]. The administration time is around slightly less than 10 min.

*Trail making test (TMT).* The TMT is a pencil-and-paper task consisting of two parts, A and B. In the first part (TMT-A), the participant is required to connect, through a pencil, 25 numbered circles in ascending order (i.e., 1–2–3 and so on), while in the second part (TMT-B), the participant is required to connect numbered (from 1 to 13) and lettered circles (from A to N) in alternated numerical vs. alphabetical ascending order (i.e., first a number and then a letter, 1–A–2–B–3–C, and so on). Participants are instructed to connect the circles as quickly as possible without taking the pencil off the paper on which the circles are printed. For both tests, the execution time (in seconds) is recorded. In particular, the time needed to complete the TMT-A is widely used as a measure of processing/psychomotor speed, visual search, and attention skills, whereas the time needed to complete the TMT-B as a measure of set-shifting and inhibition/interference control processes [88]. Overall, the TMT (TMT-A + TMT-B) takes approximately 7–10 min to be administered. In addition to TMT-A and TMT-B scores, we also calculated the B-A difference in order to remove the processing speed component from the performance on part B, thus achieving a measure of mere cognitive flexibility/set-shifting [88,92].

*Digit span backward (DSB).* The DSB is the most commonly used test in clinical neuropsychology for assessing auditory–verbal working memory (phonological loop and central executive) [93,94]. The examiner pronounces a sequence of digits (e.g., 6, 2, 9) at a rate of approximately one digit per second and the participant is asked to immediately reproduce the sequence of digits in the reverse order (e.g., 9, 2, 6). The test includes a total of six lengths (from 3 to 8 digits) and the span backward corresponds to the length of the longest list correctly repeated [89]. The DSB takes about 3 min to be completed.

*Finger tapping task (FTT)*. This task provides a quantitative assessment of gross upper-extremity motor functions [95] including speed, coordination, and rhythm [96,97]. It is considered an indirect measure of the integrity of the frontal and parietal lobes, basal ganglia, and cerebellum [62,90,98]. To perform this test, participants sat on a comfortable chair in front of a desk where a counting machine was positioned. Participants were asked to place their dominant hand palm down, fingers extended, with the index finger suspended on the screen of the digital counting device. Then, participants were instructed to repetitively tap their index finger on the counting device as quickly as possible for 10 s, being careful to keep the hand and arm stationary. Five finger-tapping trials were recorded, with a 10-s rest interval between each trial in order to decrease the effect of fatigue (average administration time = 90 s). The mean of movements recorded in the five trials (FTTm) was used in the analyses as a measure of movement speed. In the context of the present study, coordination and rhythm were not directly evaluated since we did quantify neither inter-tap intervals nor sensorimotor synchronization processes. Handedness was detected by the Edinburgh handedness inventory-short form [99]. The FTT was administered about 5 min after completion of the cognitive battery.

### 2.3. Statistical Analyses

After checking the assumptions of linear regression analysis, two four-stage hierarchical multiple regression models were constructed with BMI and WC as the dependent variables, respectively. Sociodemographic variables (i.e., sex, age, and years of education) were entered at stage 1, FAB15 was entered at stage 2, TMT (i.e., TMT-A, TMT-B, TMT(B-A)) and DSB were entered at stage 3, and FTTm was entered at stage 4. This entry order was applied, at first, to control for the confounding effects of sociodemographic variables. Then, we tested the contribution of the FAB15 score as a measure of general executive functioning. Finally, we loaded the FAB15 with additional core executive subdomains that are poorly addressed by the FAB15, i.e., set-shifting (TMT) and working memory (DSB). Lastly, FTTm entered the last block in order to assess the contribution of motor performance.

To investigate if and how motor performance mediated the relationship between executive functioning and body weight, a mediation analysis was run based on the results of the multiple regression analyses. To evaluate the significance of direct and indirect effects, bootstrap-based confidence intervals (95% CI) were constructed [100]. Statistical analyses were conducted by means of IBM SPSS Statistics v. 26. The SPSS Macro PROCESS was employed to perform the mediation analysis.

## 3. Results

### 3.1. Sample Characteristics

Data from 330 right-handed volunteers (192 females) were analyzed. Mean age was 45.98 years (SD = 17.70, range = 18–86 years) while mean education was 15.67 years (SD = 3.85, range = 5–24 years). As for anthropometric variables, mean BMI was 24.67 (SD = 3.98, range = 18.73–39.51) and mean WC was 83.35 cm (SD = 13.77, range = 58–120 cm). There were no identified differences in age, years of education or BMI between males and females (all *p*-values > 0.05); conversely, males had a larger WC compared to females (males, mean WC = 88.45, SD = 12.45; females, mean WC = 79.91, SD = 13.57; *F*_(1, 328)_ = 32.779, *p* < 0.001). Descriptive statistics on cognitive tests and FTT are summarized in Table 1.

### 3.2. Construction of Hierarchical Regression Models

For both models, relevant statistical assumptions of linear regression were tested and satisfied, i.e., the relationship between each predictor and the outcome variable was linear, no violations of the homoscedasticity principle were detected, and standardized residuals showed no significant deviation from normality based on inspection of both P–P plots and histograms. A sample size of 330 was considered adequate based on a priori power analysis (α = 0.05, 1–β = 0.80, *f*^2^ = 0.15, maximum number of predictors = 9, required sample size according to G*Power 3.1.9.4 = 114). Furthermore, we computed the variance inflation factors (VIFs) and tolerance values for each predictor in order to determine whether and how much the variance of regression coefficients could be inflated. The following rules were employed to interpret the VIF values: VIF = 1, no collinearity; VIF = 1 to 5, moderate collinearity; VIF > 5, high collinearity. Tolerance values equal to or greater than 0.10 were deemed acceptable, indicating the absence of statistically significant multicollinearity [101,102]. Unsurprisingly, when entered simultaneously into regression models, TMT-A, TMT-B, and TMT (B-A) demonstrated severe multicollinearity (VIF values > 350, tolerance values ≤ 0.003). Therefore, we decided to include only the TMT (B-A) score since it is considered a refined measure of executive control and cognitive flexibility/set-shifting [88]. *R*^2^ and *R*^2^ changes at each step in the two hierarchical regression models are reported in Table 2. The results of regression analyses are summarized in Table 3. VIF and tolerance values for the included predictors at each stage are also reported in Table 3.

### 3.3. Regression on BMI

At stage 1, when sociodemographic variables entered the regression model as predictors, a significant predictive capability (*F*_(3, 326)_ = 21.704, *p* < 0.001) was found; the model accounted for a significant amount of BMI variance (*R*^2^ = 0.22). In the following stages, the addition of the FAB15 (stage 2: *F*_(4, 325)_ = 16.498, *p* < 0.001), TMT (B-A), SDB (stage 3: *F*_(6, 323)_ = 11.122, *p* < 0.001), and FTTm (stage 4: *F*_(7, 322)_ = 9.929, *p* < 0.001) did not lead to a significant increase in the variance explained (stage 2: *R*^2^ change = 0.003, *p* = 0.34; stage 3: *R*^2^ change = 0.004, *p* = 0.36; stage 4: *R*^2^ change = 0.008, *p* = 0.06). All regression models were statistically significant for the sole contribution of sociodemographic variables.

### 3.4. Regression on WC

The hierarchical multiple linear regression revealed that, at stage 1, sociodemographic variables contributed significantly in predicting WC (*F*_(3, 326)_ = 54.895, *p* < 0.001) and explained a large proportion of its variance (*R*^2^ = 0.41). Adding the FAB15 score to the regression model (*F*_(4, 325)_ = 44.363, *p* < 0.001) explained an additional 0.02% of the variance in WC (stage 2: *R*^2^ change, *F*_(1, 325)_ = 7.967, *p* < 0.001). The addition of TMT(B-A) and SDB (*F*_(6, 323)_ = 30.151, *p* < 0.001) makes a negligible non-significant contribution to the regression model (stage 3: *R*^2^ change = 0.007, *F*_(2, 323)_ = 1.417, *p* = 0.24). Conversely, introducing the FTTm variable at stage 4 (*F*_(7, 322)_ = 28.353, *p* < 0.001), *R*^2^ significantly increased by 0.02 (*F*_(1, 322)_ = 10.378, *p* < 0.001). The final model, including all seven predictors, accounted for approximately 46% of the variance in WC. Neither TMT (B-A) (*B* = −0.01, *p* = 0.62) nor DSB (*B* = −0.84, *p* = 0.09) were significant predictors of WC.

### 3.5. Mediation Analysis

**Figure 1:**
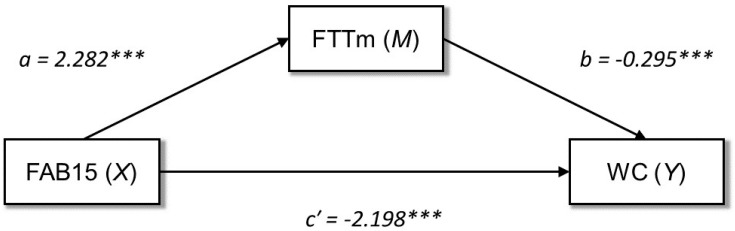
Graphical representation of the mediation analysis output. Note: FAB15: frontal assessment battery-15; FTTm: finger tapping task mean; WC: waist circumference; *a:* relationship between (X) and (M); *b:* relationship between (M) and (Y); *c′:* direct effect of (X) on (Y); *** *p* < 0.001.

Based on the results from the regression analyses, a mediation model was constructed to test the mediating role of motor performance (FTTm) in the relationship between general executive functioning (FAB15) and body fat (WC) (see Figure 1). Results indicated that FAB15 score was a significant predictor of FTTm (*a* = 2.282, 95% CI [1.392, 3.171], SE = 0.452, *t* = 5.409, *p* < 0.001). Furthermore, FTTm negatively predicted WC (*b* = −0.295, 95% CI [−0.422, −0.169], SE = 0.064, *t* = −4.600, *p* < 0.001). The indirect effect (*ab*) of FAB15 on WC through FTTm was statistically significant (*ab* = −0.674, 95% CI [−1.039, −0.332], SE = 0.181, *Z*_Sobel_ = −3.364, *p* < 0.001). Since the estimated direct effect (controlled for the mediator) of FAB15 on WC was still significant (*c′* = −2.198, 95% CI [−3.197, −1–200], SE = 0.507, *t* = −4.335, *p* < 0.001), motor performance partially mediated the relationship between general executive functioning and body fat. Specifically, FAB15 showed an indirect negative effect on WC partially conveyed by its positive association with FTTm. Thus, a higher executive functioning predicted a decrease in WC explained, in part, by the positive linear relationship between EFs and motor performance.

## 4. Discussion

In the present cross-sectional study, we investigated the relationship between EFs, motor skills, and body weight in a sample of healthy individuals from the general population. In particular, we firstly tested the hypothesis that EFs better explained the variance of WC than BMI. Subsequently, we explored the mediating role of motor skills in the relationship between EFs and body weight. In line with our expectations, we found that WC is a more effective measure for studying the association between EFs and body weight as compared to BMI. Still, we found that the motor domain played a non-negligible role in the dynamics of such a relationship. In a scientific context in which the link between EFs and obesity needs to be further investigated [11,16,23,24,25,103,104], these findings may contribute to extending the debate on the matter.

Variability in WC (visceral body fat) was explained by both general executive functioning (FAB15) and motor skills (FTT). More specifically, our results showed that WC displayed an inverse relationship with both the FAB15 score, which covers different executive domains, such as abstraction abilities, generativity, planning, susceptibility to interference, and inhibitory control [87], and the FTTm score, a measure of upper limb gross motor function [95]. In addition, results from the mediation analysis highlighted that FTTm score partially mediated the relationship between general executive functioning and visceral body fat. The indirect effect of FAB15 on WC through FTTm is likely the result of the activity of different brain regions involving frontal and parietal lobes, basal ganglia, and cerebellum [62,90,98]. According to the sensorimotor interaction model of behavior [60], sensorimotor integration processes guarantee the development of action control abilities via interaction with the environment, which enhances, in turn, the maturation and efficiency of cognitive processes [61]. In line with this vein, noteworthy is the role of the cerebellum that assists in the adjustment and automation of movements according to environmental demands and contextual changes for promoting adaptive behaviors. It is worthy of equal interest the anatomo-functional interaction between the occipito-temporal cortex and the reward centers in the basal ganglia and prefrontal cortex. The activity of this network contributes to evaluating the reward value of an object and anticipating/predicting reward-related expectancies and outcomes of the action [61]. In line with these claims, it might be misleading to ascribe EFs to the sole prefrontal cortex. Indeed, EFs are also related to sensorimotor circuits responsible for processing sensory information, associating this information with reward values, and programming and executing appropriate actions.

Neuroimaging evidence has suggested that obese subjects show decreased cortical thickness in the superior frontal gyrus and medial orbitofrontal cortex; in these patients, the volumes of the ventral diencephalon, brainstem [105], and cerebellum [106] also appear to be reduced. Moreover, obese patients would show decreased neural plasticity within the frontal cortex [84]. These findings tend to suggest that obese patients may show reduced sensorimotor integration processes, which are strictly related to visuomotor and cognitive processes.

The visuomotor system uses spatial representations generated within the fronto-parieto-cerebellar network, in which the occipitoparietal/dorsal stream and cerebellum play a crucial role [107,108,109,110,111]. The dorsal stream, combining retinotopic and spatiotopic/body-centered coordinates, supports the planning and execution of real-time visually-guided actions [112,113,114,115,116,117,118]. The cerebellum is responsible for the muscular coordination of the distal portions of the limbs, with particular reference to hands and digits. It guarantees harmonious progression and timing of every single movement during the execution of coordinated motor sequences. The cerebellum is the structural core of the feedforward network involved in the predictive phase of the action [119,120,121]. Before starting a movement, the cerebellum receives a copy of the motor command from the frontal cortex and sensory inputs from the parietal cortex [122]. Based on sensory feedback (i.e., vestibular and proprioceptive signals) from the distal body parts, the cerebellum executes a kind of “quality control” and can send rapid corrective signals to the neocortex and spinal cord [62]. Importantly, the cerebellum acts in concert with the nigrostriatal pathway including the substantia nigra pars compacta and the dorsal striatum. The substantia nigra pars compacta supply dopamine to the striatum [123], which enhances—via the frontal cortex—adaptive motor programs and higher cognitive control of hand movements [62,124].

To further disentangle the relationship between visceral body fat and cognition, the functional interaction between body weight and brain physiology needs to be taken into account. There is speculation in the literature on the role exerted by inflammatory processes.

Higher levels of white adipose tissue might increase systemic inflammation. It is widely acknowledged that adipocytes, lymphocytes, and macrophages lead to the production of pro-inflammatory cytokines [125,126,127,128,129,130,131]. In addition, a diet high in fatty acids and sugar may undermine the integrity of the blood–brain barrier [29], which plays a key role in protecting the central nervous system from bloodborne toxins.

Some studies have shown that central inflammation is often observed after high-fat feeding, especially in the hypothalamus region [2,132,133,134,135], and might predict cognitive decline and/or the onset of major neurocognitive disorders [30,31,32,33]. Interestingly, adipose tissue may modify β-amyloid metabolism [136,137]. Indeed, some neuropathological features of Alzheimer’s disease, such as amyloid plaques and neurofibrillary tangles, appear to be most observed in elderly obese people as compared to normal-weight older adults [138]. Finally, a relationship between midlife obesity, blood–brain barrier dysfunction, and primary and/or secondary dementias, has been hypothesized [28,139].

The main limitation of the current study is its cross-sectional nature, which does not allow to draw conclusions about causality.

## 5. Conclusions

Our results highlight a relationship between general executive functioning and visceral body fat, with the gross motor function, which partially mediates such a relationship. The indirect effects of EFs on body fat through motor function may be explained by shared neural substrates mainly embracing the fronto-parieto-cerebellar network. Since most of the tasks assessing EFs in both clinical and experimental practices require a motor response to be performed, future studies could further investigate executive performance in the obese population by administering both motor- and non-motor-based executive tasks.

## Figures and Tables

**Table 1:** Descriptive statistics of the neurocognitive measures employed.

	Mean	SD	Range
MMSE ^a^	29.21	1.22	23–30
FAB15 ^a^	13.63	1.54	7–15
TMT-A, seconds ^a^	37.89	18.79	15–100
TMT-B, seconds ^a^	95.10	42.53	20–239
TMT(B-A), seconds ^a^	57.91	31.38	2–151
DSB ^a^	6.05	1.43	4–8
FTTm	63.42	12.23	16.80–91.40

MMSE: Mini-mental state examination; FAB15: frontal assessment battery-15; TMT-A: trail making test-A; TMT-B: trail making test-B; TMT (B-A): trail making test (B-A); DSB: digit span backward; FTTm: finger tapping task mean; ^a^ raw scores.

**Table 2:** *R*^2^ change at each step hierarchical regressions on body mass index and waist circumference.

	Predictors	*R*^2^ for Model	*F* For Model	*R*^2^ Change	*F* for *R*^2^ Change
	Outcome: BMI				
1	Sex, age, education	0.223	*F*(3, 326) = 21.704 ***	0.223	*F*(3, 326) = 21.704 ***
2	Sex, age, education, FAB15	0.226	*F*(4, 325) = 16.498 ***	0.003	*F*(1, 325) = 0.906
3	Sex, age, education, FAB15, TMT(B-A), DSB	0.230	*F*(6, 323) = 11.122 ***	0.004	*F*(2, 323) = 0.512
4	Sex, age, education, FAB15, TMT(B-A), DSB, FTTm	0.238	*F*(7, 322) = 9.929 ***	0.008	*F*(1, 322) = 0.126
	Outcome: WC				
1	Sex, age, education	0.408	*F*(3, 326) = 54.895 ***	0.408	*F*(3, 326) = 54.895 ***
2	Sex, age, education, FAB15	0.427	*F*(4, 325) = 44.363 ***	0.019	*F*(1, 325) = 7.967 ***
3	Sex, age, education, FAB15, TMT(B-A), DSB	0.434	*F*(6, 323) = 30.151 ***	0.007	*F*(2, 323) = 1.417
4	Sex, age, education, FAB15, TMT(B-A), DSB, FTTm	0.458	*F*(7, 322) = 28.353 ***	0.024	*F*(1, 322) = 10.378 ***

BMI: body mass index; FAB15: frontal assessment battery-15; TMT (B-A): trail making test (B-A); DSB: digit span backward; FTTm: finger tapping task mean; WC: waist circumference. *** *p* < 0.001.

**Table 3:** Results of the hierarchical regression analysis on BMI and WC.

	Predictors	*B*	95% CI for *B*		*SE*	*t*	*p*-Value	VIF	Tol.
			**LL**	**UL**					
Outcome:	BMI								
Stage 1	Sex	−1.533	−2.363	−0.703	0.421	−3.640	**<0.001**	0.991	1.009
	Age	0.061	0.036	0.086	0.013	4.834	**<0.001**	0.990	1.010
	Education	−0.323	−0.451	−0.196	0.065	−4.994	**<0.001**	0.991	1.009
Stage 2	Sex	−1.564	−2.396	−0.731	0.423	−3.701	**<0.001**	0.985	1.015
	Age	0.066	0.039	0.093	0.014	4.812	**<0.001**	0.833	1.200
	Education	−0.351	−0.490	−0.211	0.071	−4.950	**<0.001**	0.829	1.207
	FAB15	−0.175	−0.158	0.539	0.184	−0.952	0.342	0.704	1.420
Stage 3	Sex	−1.585	−2.421	−0.750	0.424	−3.738	**<0.001**	0.982	1.018
	Age	0.060	0.030	0.090	0.015	3.897	**<0.001**	0.669	1.494
	Education	−0.327	−0.479	−0.176	0.077	−4.260	**<0.001**	0.708	1.412
	FAB15	−0.221	−0.168	0.610	0.197	−1.119	0.264	0.617	1.621
	TMT(B-A)	0.006	−0.010	0.021	0.008	0.727	0.468	0.685	1.459
	DSB	−0.126	−0.465	0.213	0.172	−0.731	0.466	0.759	1.318
Stage 4	Sex	−1.945	−2.897	−0.993	0.483	−4.025	**<0.001**	0.752	1.329
	Age	0.050	0.017	0.083	0.017	3.025	**0.003**	0.572	1.750
	Education	−0.326	−0.477	−0.175	0.077	−4.257	**<0.001**	0.708	1.412
	FAB15	−0.215	−0.173	0.603	0.197	−1.091	0.276	0.617	1.622
	TMT(B-A)	0.004	−0.012	0.019	0.008	0.460	0.646	0.666	1.502
	DSB	−0.116	−0.454	0.223	0.172	−0.673	0.502	0.758	1.320
	FTTm	−0.042	−0.095	0.012	0.027	−1.538	0.126	0.568	1.760
Outcome:	WC								
Stage 1	Sex	−10.369	−12.831	−7.907	1.250	−8.296	**<0.001**	1.007	0.993
	Age	0.294	0.221	0.367	0.037	7.937	**<0.001**	1.003	0.997
	Education	−0.988	−1.377	−0.600	0.197	−5.011	**<0.001**	1.008	0.992
Stage 2	Sex	−10.465	−12.893	−8.037	1.233	−8.491	**<0.001**	1.008	0.992
	Age	0.334	0.257	0.412	0.039	8.527	**<0.001**	1.155	0.866
	Education	−1.233	−1.653	−0.814	0.213	−5.793	**<0.001**	1.209	0.827
	FAB15	−1.503	−2.553	−0.454	0.533	−2.823	**0.005**	1.371	0.729
Stage 3	Sex	−10.486	−12.924	−8.049	1.237	−8.477	**<0.001**	1.019	0.982
	Age	0.325	0.238	0.412	0.044	7.349	**<0.001**	1.469	0.681
	Education	−1.217	−1.676	−0.758	0.233	−5.221	**<0.001**	1.454	0.688
	FAB15	−1.827	−2.942	−0.712	0.566	−3.229	**0.001**	1.553	0.644
	TMT(B-A)	−0.003	−0.049	0.043	0.023	−0.130	0.897	1.502	0.666
	DSB	−0.848	−1.856	0.161	0.512	−1.657	0.099	1.335	0.749
Stage 4	Sex	−12.642	−15.371	−9.912	1.385	−9.125	**<0.001**	1.328	0.753
	Age	0.254	0.159	0.350	0.048	5.249	**<0.001**	1.841	0.543
	Education	−1.168	−1.619	−0.717	0.229	−5.099	**<0.001**	1.461	0.685
	FAB15	−1.746	−2.841	−0.652	0.556	−3.144	**0.002**	1.556	0.643
	TMT(B-A)	−0.011	−0.057	0.034	0.023	−0.494	0.622	1.521	0.658
	DSB	−0.840	−1.829	0.149	0.502	−1.673	0.096	1.335	0.749
	FTTm	−0.255	−0.410	−0.099	0.079	−3.221	**0.001**	1.788	0.559

BMI: body mass index; FAB15: frontal assessment battery-15; TMT (B-A): trail making test (B-A); DSB: digit span backward; FTTm: finger tapping task mean; WC: waist circumference.

## Data Availability

The data presented in this study are available upon request from the corresponding author.
